# The Practitioner: Reputed and Actual

**Published:** 1910-12-24

**Authors:** 


### SPECIAL ARTICLES.

### THE PRACTITIONER: REPUTED AND ACTUAL.

When the ordinary business man or stockbroker wishes to speak nicely to a medical friend, he is prone to remark: "I shouldn't have been clever enough for a doctor"; or "I should never have had the nerve for operations." Such compliments, though often sincere enough, have little behind them in the way of fact. What the speaker, knowingly or unknowingly, would convey, is a recognition that although a learned profession may not pay very well, yet to have entered one is some little achievement, and to have knowledge of the appropriate subject-matter is intellectually elevating. There he is quite right; the medical curriculum must improve the dullest, the most snobbish, the most conventional of minds. But originally the boy destined to be a doctor (setting aside some scholarship winners and a few of distinct scientific bent) is not any brighter than the one articled to a solicitor or the one taken into a counting-house. The great bulk of a year's entry of medical students find themselves where they are as a result of the weighing of economic considerations by their parents; for in spite of grumbling about the cost of medical education, the public knows that ours is the only profession in which a man has but to be well behaved to be perfectly sure of his bread and butter. Art alone obtains solely votaries with some degree of inborn aptitude, a fact noted with characteristic perspicacity by Galton, who points out that the eminence of great artists is eminence of an exceedingly high grade, because art students are themselves a selected class. However, there must be qualities which a medical career especially demands, and it is interesting (interesting not only because, as Peer Gynt said of the Church's funeral orations, the topic is "immovably oneself") to learn, on the authority of our great critic the laity, that the said qualities are intellectuality and courage.

It is interesting, indeed, but, as already hinted, not quite convincing, although plenty of medical men share the opinion, at all events the first part of it. How very many go through life honestly thinking themselves intellectual men just because they have obtained an M.D. or an F.R.C.S.! This too when, year in, year out, the highest forms of recreation they seek are the daily paper (and not much of that), a musical comedy now and again, and fretwork puzzles! And why do clinicians delude themselves with the idea that the ratiocination called for in making a physical examination is of a high order? Have they never seen a good engineer or plumber deducing what is wrong with a machine or where the fault is in a wire? In the former case the object is greatly more complex, it is true, but still the class of reasoning is just the same, and the knowledge upon which that reasoning is based infinitely more imperfect. Again, the problem of tackling big text-books successfully enough to pass an examination in them is much more a moral than an intellectual one; nowadays every industrious bank clerk has text-books, which he makes good use of, and so have higher class mechanics. As for the use of scientific apparatus, that is a natural gift quite independent of intellectual ability, just as in the artistic sphere technique is quite independent of temperament. Capital bacteriologists in the rough have been made, as officers of the Indian Medical Service have recounted before now, out of Asiatic gaol prisoners; the real authority on analysis, public health students sometimes find, is the humble laboratory attendant. In fine, it is just a matter of experience that to a student minus intellectual ability but possessed of ambition, common sense, correct social antecedents, an independent income, and a pleasant manner, even a place on a metropolitan hospital teaching staff is easily accessible.

As to courage, happily, we may go further with our complimentary critic, though still not so far as he would have us go. The current Medical Directory gives the number of British qualified practitioners as forty thousand odd. How many of these habitually perform major operations? A quarter, perhaps; hardly more. It may be objected that performing operations is not the only test of courage. If a medical man dare not run the risk of killing his patient, it may be said, he has still to face the chances of infection, and he must keep undismayed in his often losing fight with every form of disease. This is so, and indeed in the medical life two sorts of courage are needed, corresponding somewhat to the two sorts of justice described by Aristotle; these are courage in one's own self-conduct—the courage of self-exposure to risk—and courage displayed in dealings with one's fellows—the courage of antagonism. A medical man's need of the first kind will naturally suggest itself, as we have seen, to the intelligent layman. But the laity greatly exaggerates the chief risk, that of infection with disease, and therefore gives doctors too much credit for bravery, the real truth being that the navvy's risk of accident is a good deal more serious. However, the danger of contracting malignant scarlatina from a patient, or septicæmia from performing an autopsy, is not exactly imaginary, and there are practitioners who show a quiet but rather keen appreciation of this fact, while in other respects they are more intrepid and aggressive than their fellows. The man who is not fond of lingering near a diphtheria case, who avoids surgery and even contrives that another hand shall push in the aspirating needle, is by no means necessarily a person who can be slighted with impunity. He may very easily be of considerable force of character, one whom patients obey instinctively, one who is satisfied with nothing less than consultant rank, a dominating speaker at medical meetings. And conversely the laboratory worker who will handle plague cultures nonchalantly or even carelessly, or the young and jealous surgeon who will rush in—and likely fail—where better men fear to operate, may each of them be intimidated by adverse companions or circumstances. The first has the courage of antagonism, the other two of self-exposure to risk—very different qualities, it would appear, since each is associated with a distinct temperament, and while the first increases up to the sixth decade, the other is at its height in youth. The medical man needs the former as well as the latter, not only to help him succeed in the competitive struggle which goes on in a still overcrowded profession, but also that he may keep his patient obedient to him, and preserve a good conceit of himself in the conduct of those sadly numerous cases in which, medical therapeutics being what they are, he can do practically nothing.

But the complete virtue is possessed by few men of any sort or condition; and no reason exists for supposing that there are more of them in medicine than in any other profession. The present writer once heard it agreed that doctors, when they undertook anything, were less easily daunted than laymen; but then, unfortunately, the originator of the opinion was a medical man himself, and though eminent in his day, his eminence had been shown only on the international football field. Of course, the number of our completely brave men who have a fine intellect as well is small indeed. Of such a paragon—an Ambroise Paré or a John Hunter—it must be confessed, in the words of Peacock's pleasant song, that

"... Nature had but little clay
Like that of which she moulded him."

